# Supplementary material for: Influence of Laparoscopic Surgery on Cellular Immunity in Colorectal Cancer: A Systematic Review and Meta-Analysis
Source: Cancers (Basel). 2023 Jun 28;15(13):3381. doi: 10.3390/cancers15133381 (PMC10340378; doi:10.3390/cancers15133381)
Supplement: Supplementary file 1 [file cancers-15-03381-s001.zip › cancers-2419623-supplementary figures.pdf]

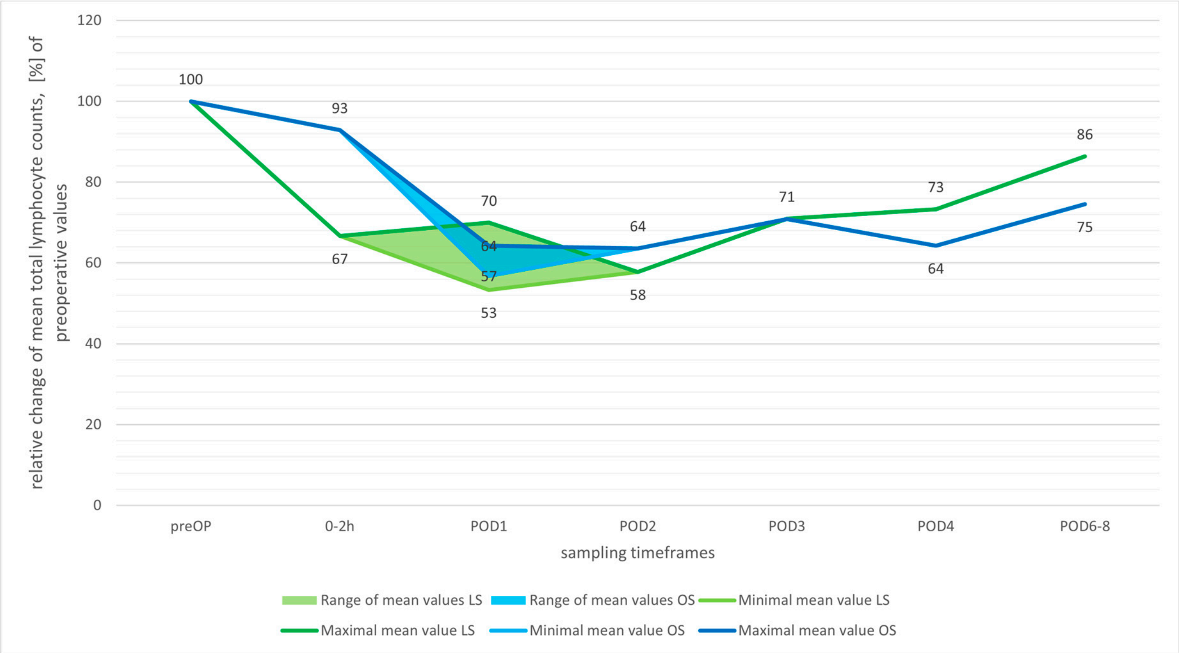

Figure S1. Postoperative development of total lymphocyte count relative to preoperative measurements, relative change in means with preoperative values set at 100%.

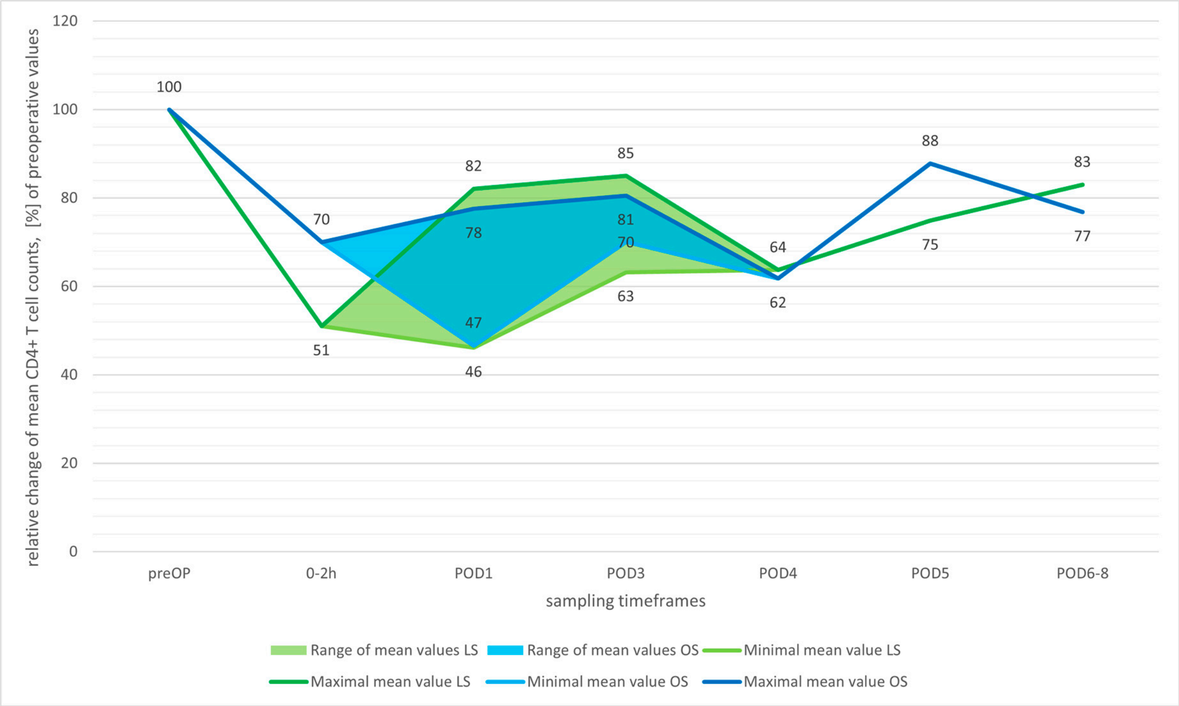

Figure S2. Postoperative development of CD4+ T cell count relative to preoperative measurements, relative change in means with preoperative values set at 100%.

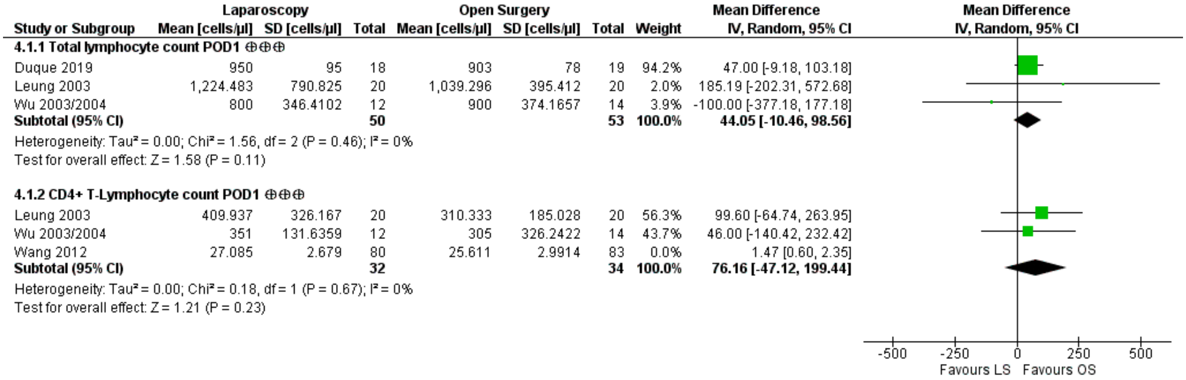

Figure S3. Forest plot of total lymphocyte and CD4+ T lymphocyte counts at postoperative day (POD)1; ⊕⊕⊕ indicates moderate confidence in estimates of effect [50,55,60–62].

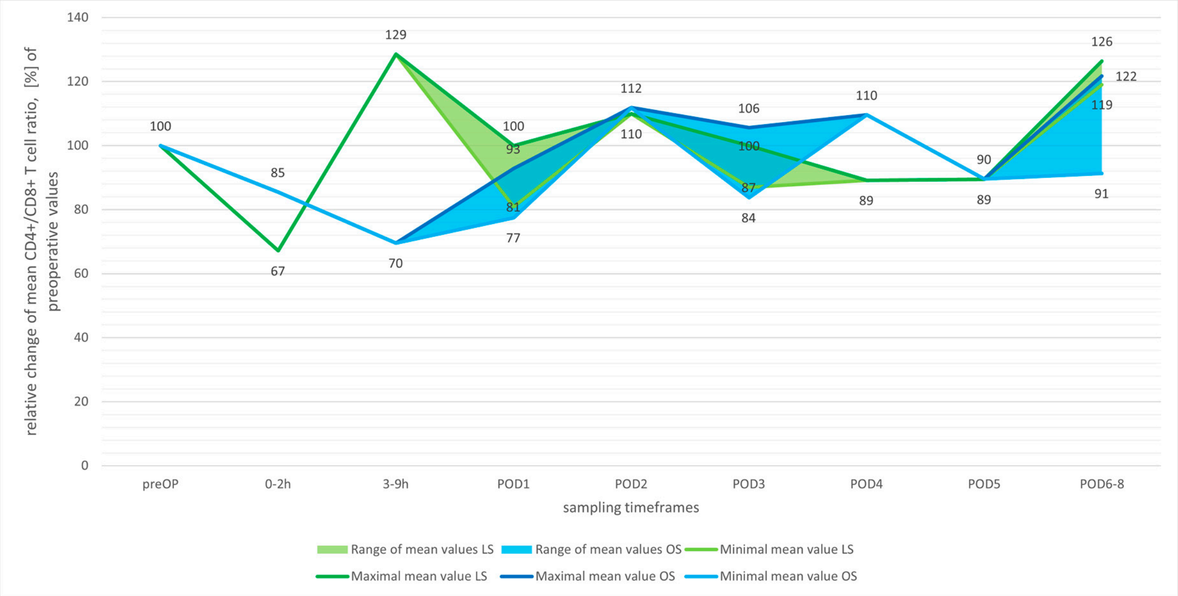

Figure S4. Postoperative development of CD4+/CD8+ ratio relative to preoperative measurements, relative change in means with preoperative values set at 100%.

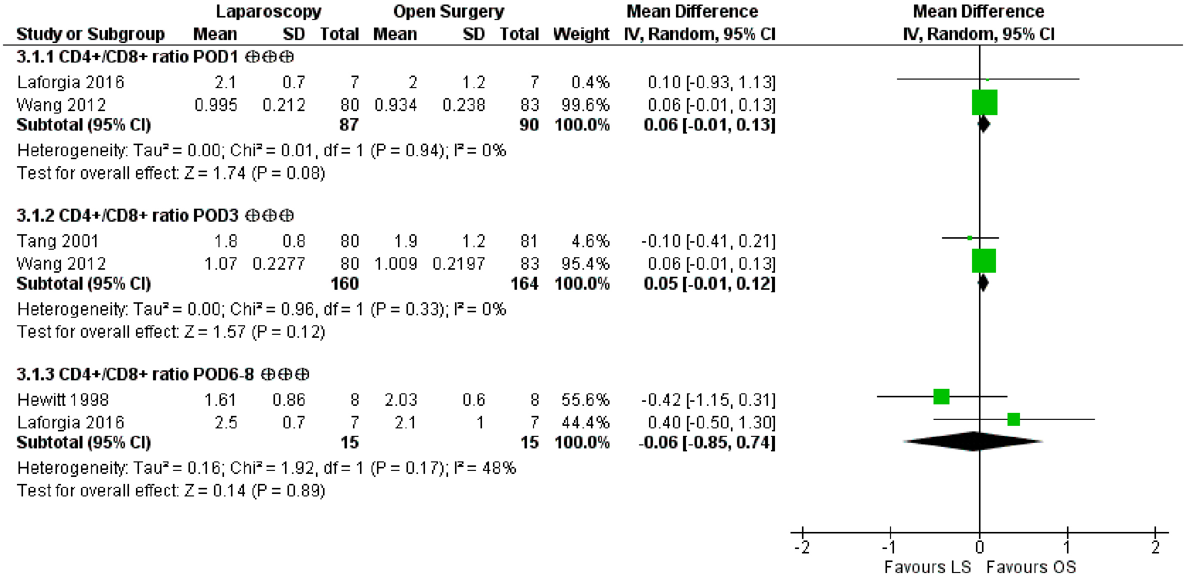

Figure S5. Forest plot of CD4+/CD8+ ratio at postoperative day (POD)1, POD3, POD6–8; ⊕⊕⊕ indicates moderate confidence in estimates of effect [48,49,52,60].
